# Does the Cognitive Reflection Test Work with Chinese College Students? Evidence from a Time-Limited Study

**DOI:** 10.3390/bs14040348

**Published:** 2024-04-22

**Authors:** Zhaoxian Li, Shangsong Yan, Jie Liu, Wei Bao, Junlong Luo

**Affiliations:** 1 School of Psychology, Shanghai Normal University, Shanghai 200234, China; 1000513619@smail.shnu.edu.cn (Z.L.); 1000477918@smail.shnu.edu.cn (J.L.);; 2 Lab for Educational Big Data and Policymaking, Ministry of Education, Shanghai Normal University, Shanghai 200234, China

**Keywords:** cognitive reflection test, intuition, analytical thinking, time limited, Chinese college students

## Abstract

The cognitive reflection test (CRT) is an experiment task commonly used in Western countries to test intuitive and analytical thinking styles. However, the validity of this task for Chinese participants has not been explored. Therefore, this study recruited Chinese college students to finish CRT tasks with various experimental designs. To gauge the accuracy of the CRT tasks, 438 Chinese college students first completed online questionnaires. Participants were then invited to participate in an offline laboratory with the same experimental settings. Finally, time pressure was used to strictly control intuition and analytical thinking to explore the performance of Chinese college students on CRT tasks. The results show that of the three experiments, Chinese college students had the highest accuracy in the offline test, and the CRT’s intuitive conflict problem still applies to Chinese students under the time-limited condition. This study demonstrates the validity of the CRT in China and proves that time pressure is an effective method for identifying individuals with strong logic ability.

## 1. Introduction

The renowned cognitive reflection test (CRT), first proposed by Frederick (2005) [1], is utilized to measure individual differences in intuitive-analytic cognitive style and as research material for the dual-process theory. The most significant characteristic of this test is that most people can easily and effortlessly provide intuitive but incorrect answers [2], while the correct answers require cognitive resources, the inhibition of initial responses [3], and the use of some simple mathematical calculations [4,5,6].

According to the dual-processing theory of thinking, System 1 is supposed to be intuitive thinking and is assumed to operate quickly and effortlessly [7]. It is System 1 that guides us to answer 10 cents to the first question. System 2 is thought to be slower and more laborious because using it burdens our limited cognitive resources. System 2 is believed to regulate the type of deliberate thinking that is usually required for sound logical reasoning. Frederick identified the possibility that cognitive reflexes are a tendency to think. Arriving at the right answer to CRT requires the use of sound reasoning to shift thinking to more critical deliberation and correct the initial intuitive response. This is explained using the default-interventionist conception of System 2 [8], which argues that errors in the CRT are caused by the failure of System 2 to monitor or override System 1’s functioning. Reasoners that rely on System 1 have a strong tendency to minimize demanding calculations, and many reasoners avoid engaging in or completing such laborious deliberations [9]. In addition, Campitelli and Labollita (2010) [10] proposed that cognitive reflection is not only an ability or disposition to veto a prepotent response but also an ability or disposition to initiate cognitive processes. This ability should not considered a general cognitive ability (e.g., intelligence, working memory), but rational thinking [2]. This kind of rational thinking, in fact, requires deliberate thought; another characteristic is the high demand of our limited cognitive resources [3,11]. Individuals with high cognitive ability are more likely to have the necessary resources and/or motivation to complete a thoughtful process and correct their faulty intuitions. 

Furthermore, in addition to measuring cognitive tendencies and cognitive abilities, most scholars believe that CRT problems are distinct from other mathematical problems in that they trigger an automatic response, which is then either inhibited or not inhibited [12]. Only when inhibition is successful do individuals utilize their mathematical knowledge to solve the problem [4]. In CRT solving, if an individual realizes that the intuitive answer is not the right answer, then finding the right answer requires relatively simple mathematical calculation. Weller et al. (2013) [13] included two CRT problems within their numeracy scale, and they discussed the CRT within a section entitled “Existing measures of numeracy.” In this way, they meant that the CRT is just a test of mathematical ability. While the math required for each problem is neither complex nor difficult, it is easy to fall into the trap of “intuition”. In the same way, Campitelli and Gerrans (2014) [4] used mathematical modeling to find that women’s performance in the CRT in females is accounted for by their abilities (both mathematical and rational thinking abilities). On the basis of these observations, it can be concluded that addressing CRT problems continues to necessitate elementary mathematical operations with mathematical proficiency, potentially exerting a certain degree of influence.

Large-scale cross-country comparative studies of mathematical abilities [14,15,16] show that East Asian countries, such as China, Japan, South Korea, and Singapore, generally lead in mathematical performance. These country have also achieved notable results in international mathematics competitions and assessments [17]. In addition, East Asians have an advantage in math scores across multiple age groups, starting with preschool children [18,19]. In addition to the different educational methods employed in the region, East Asian pupils often devote more hours per day to mathematics (among other academic subjects) in school and in homework than those in many other countries [20]. Hence, it is possible that Asians may have a slight advantage in terms of mathematical abilities, yet the performance of CRT among Asian university students remains uncertain. 

Although the math required is neither complicated nor difficult, people tend to perform poorly on the CRT. Many Western scholars use the bat-and-ball problem from the CRT as experimental material to explore intuition and analytical thinking. After reviewing the literature, we found that the accuracy of CRT in Western countries is less than 40%. De Neys et al. (2013) [21] found that the rate of correct answers to an adapted bat-and-ball problem was the same as that of the standard one, with only 21% of participants answering correctly. In Bago et al.’s three studies (2019) [22], the accuracy rates for the bat-and-ball problem were only 27.3%, 17.8%, and 23.8%, respectively. Boissin et al. (2021) [23] used a dual-response paradigm, requiring participants to provide an initial answer to the CRT task, followed by a final answer. The results showed that the accuracy rate of the two answers was not high when the conflicts were not explained to the participants. Table 1 is a summary of experimental studies using the CRT’s bat-and-ball problem. In the same way, a meta-analysis of CRT by Branas-Garza [24] also found that 32% of 116 studies were correct about bat-and-ball questions. The correct resolution of CRT tasks does not necessarily indicate an individual’s mathematical proficiency, but it still involves a certain level of mathematical operations. However, we do not yet know how college students growing up in different educational backgrounds perform on the CRT. That is, how East Asian college students perform on the CRT remains an open question. This is one of the main questions to be explored in this study.

CRT is not solely a test of mathematical ability. Individuals only utilize their mathematical knowledge to solve problems when the inhibition of their initial response is successful. Time pressure, in the form of a response deadline, plays a crucial role in inhibiting the “initial response” [31,32]. Generally, short response deadlines decrease both the response process and accuracy in thinking tasks. In studies on intuition and analytical thinking, cognitive load [33] or time pressure are usually used to control participants’ intuitive answers. Specifically, Johnson et al. (2016) [28] asked participants to solve the bat-and-ball problem under different cognitive loads and found that the correct response rates were only 21.6%, 15.9%, 3.3%, and 3.3% under no cognitive load, low load, high load, and extra-high load, respectively. In addition to controlling cognitive load to ensure that individuals use intuitive thinking, reaction time pressure is another way to ensure intuitive thinking. Evans and Curtis-Holmes (2005) [34] compared participants’ performance with and without time constraints by controlling response times for four types of syllogism reasoning tasks (valid trust, valid untrust, invalid trust, and invalid untrust). The results showed that compared with the group without a time limit, belief bias responses increased and logical responses decreased in the time-limited group. This evidence suggests a subtle and inextricable relationship between time pressure and whether individuals give intuitively incorrect or analytically correct answers. Evans (2006) [8] pointed out that heuristic thinking tends to produce an initial default bias response. Analytical thinking may intervene to modify this process, but it often depends on factors such as the individual’s cognitive ability and time availability. Studies have shown that when participants are asked to respond quickly, they sacrifice accuracy by lowering their decision thresholds and making choices based on less evidence [35,36,37]. 

In addition to time pressure and cognitive load that can affect an individual’s intuitive and analytical thinking processes, the experimental environment may also have an impact. The collection of online data is increasing, with its greatest advantage being the ability to collect data on a large scale quickly. However, the quality of the data may decline, especially in the case of the CRT, which involves intuitive traps. Studies have found that online learning feels like less pressure than face-to-face interactions [38,39,40]. The environmental pressure here is different from time pressure. Moderate pressure promotes cognition [41,42], while the relaxed environment online may conversely lead individuals to make relatively simple intuitive errors. Therefore, we hypothesize that the CRT performance may differ between online and offline experiments.

In summary, CRT serves as a pivotal assessment tool for evaluating cognitive thinking patterns, with the capacity to induce erroneous intuitions. Its successful resolution necessitates both the inhibition of initial responses and the intervention of mathematical operations to arrive at the correct answers. Moreover, the smooth execution of CRT tasks is susceptible to factors such as time pressure and cognitive load. Variations in mathematical pedagogy and approaches across certain Asian countries have contributed to a slight advantage in mathematical proficiency among Asian students. Nevertheless, the performance of this demographic on CRT tasks remains uncertain. In the current study, three experiments are conducted to validate previous studies. Chinese college students were used in the experiment to explore whether the CRT for detecting intuitive conflict remains applicable in China.

## 2. Experiment 1

In this experiment, we initially conducted a large-scale online survey of the cognitive reflection test (CRT). The aim was to preliminarily explore the performance of individuals under the Chinese educational background in the CRT. The CRT answers were collected through an online questionnaire, which is easy and convenient, without any pressure. We hypothesize that this survey may yield results similar to those in Western countries.

### 2.1. Method

#### 2.1.1. Participants

Participants were recruited online, using the Academic Questionnaire Website (https://www.wjx.cn/, accessed on 1 December 2021). A total of 438 participants (126 males, mean age = 21.40 ± 2.44 years) were recruited (all gave written informed consent). Most participants reported a bachelor’s degree (84.5%) as the highest completed level of education, followed by a master’s degree (11.4%) and high school (4.1%), respectively.

#### 2.1.2. Materials

Taken from Frederick’s study (2005) [1], this test was composed of three questions. To ensure the that the unit of calculation was appropriate for China, we changed the currency from the USD to the CNY.

1. A bat and a ball cost $1.10 in total. The bat costs $1.00 more than the ball. How much does the ball cost?
2. If it takes 5 machines 5 min to make 5 widgets, how long would it take 100 machines to make 100 widgets?
3. In a lake, there is a patch of lily pads. Every day, the patch doubles in size. If it takes 48 days for the patch to cover the entire lake, how long would it take for the patch to cover half the lake?

#### 2.1.3. Procedure

The experiment was run online through the Questionnaire platform. Participants were specifically told that the experiment would demand their full attention throughout. After presenting a series of irrelevant questions, the participants were presented with the CRT questions, which the participants were required to answer in a limited amount of time. Due to the specificity of the CRT, once the rules within the questions are understood, it becomes challenging to fall into intuitive traps. In other words, prior exposure to CRT significantly enhances test performance [43,44,45]. Therefore, at the conclusion of the experiment, participants are typically asked whether they have encountered such questions before. After completing the task, the participants were randomly given a reward of two to four CNY.

### 2.2. Results

Table 2 shows the accuracy rate for each question in the CRT. We conducted a chi-square test to analyze the relationship between gender and CRT score and found a significant correlation between gender and CRT scores (bat-and-ball: *χ*^2^(1, 438) = 8.326, *p* = 0.004, Cramer’s V = 0.138; machine: *χ*^2^(1, 438) = 10.932, *p* = 0.001, Cramer’s V = 0.158; lily pads: *χ*^2^(1, 438) = 23.523, *p* = 0.000, Cramer’s V = 0.232). The Mantel–Haenszel chi-square test was used and found no significant correlation between education level and CRT scores (bat-and-ball: MH*χ*^2^(4) = 7.637, *p* = 0.106; machine: MH*χ*^2^(4) = 7.827, *p* = 0.098; lily pads: MH*χ*^2^(4) = 2.832, *p* = 0.586). A correlation analysis with point-biserial correlation found no significant correlation between age and CRT score (bat-and-ball: *r* = 0.78, *p* = 0.102; machine: *r* = 0.025, *p* = 0.597; lily pads: *r* = 0.001, *p* = 0.991).

### 2.3. Discussion

In the correlational analysis, it was found that CRT is significantly related to gender, with males scoring higher on the CRT than females. There is no significant correlation between education level and CRT score, which indicates that an individual’s cognitive thinking tendency is not entirely dependent on education level. This is consistent with previous research findings [1,46]. However, the accuracy rate of our CRT was 68.5%, which was slightly higher than that of previous studies.

## 3. Experiment 2

In Experiment 1, we obtained results similar to previous studies on CRT. In Experiment 2, we invited college student participants to the laboratory to complete the CRT, aiming to validate and replicate the results of Experiment 1 in a laboratory setting.

### 3.1. Method

#### 3.1.1. Participants

A total of 120 participants (48 males, mean age = 21.225 ± 2.833 years) were recruited from Shanghai Normal University. All the participants were students from the first year of college to the second year of graduate school. This experiment was approved by the local ethics committee. All participants provided written informed consent in accordance with the Declaration of Helsinki [47] and received a monetary reward for their participation in the experiment.

#### 3.1.2. Procedure

Experiment 2 used the same material as Experiment 1.

After the participants came to the laboratory and completed the informed consent, they first responded to an unrelated procedure. Participants were then given a paper with the CRT task printed on it and asked to write down their answers. After the experiment was completed, the participants received 10 CNY.

### 3.2. Results

Table 2 shows the accuracy rate for each question in the CRT.

We utilized a chi-square test and discovered a significant correlation between gender and CRT scores (bat-and-ball: *χ*^2^(1, 120) = 4.123, *p* = 0.042, Cramer’s V = 0.185; machine: *χ*^2^(1, 120) = 1.911, *p* = 0.167, Cramer’s V = 0.126; lily pads: *χ*^2^(1, 120) = 8.024, *p* = 0.005, Cramer’s V = 0.259). Mantel–Haenszel chi-square was used to analyze grade and CRT scores and found that there were significant differences between grade and CRT in machine and lily pads (bat-and-ball: MH*χ*^2^(1) = 1.935, *p* = 0.164; machine: MH*χ*^2^(1) = 8.849, *p* = 0.003; lily pads: MH*χ*^2^(1) = 4.009, *p* = 0.045). Point-biserial correlation analysis found no significant correlation between age and CRT score, and age is not correlated with CRT scores (bat-and-ball: *r* = −0.077, *p* = 0.401; machine: *r* = −0.176, *p* = 0.062; lily pads: *r* = −0.145, *p* = 0.113).

### 3.3. Discussion

In Experiment 2, the performance of the CRT in the laboratory environment showed an upward trend, reaching 87%. In the CRT, the bat-and-ball and lily pads still showed a significant correlation with gender, with males performing better than females. Furthermore, the analysis related to grade level revealed that lower-grade university individuals performed better on the machine and lily pads than higher-grade individuals. We speculate that this may be due to the fact that lower-grade individuals are better able to simulate exam conditions in an offline experimental environment, exhibiting a state of alertness under exam conditions.

## 4. Experiment 3

In Experiments 1 and 2, participants had high response accuracy for the bat-and-ball problem. In Experiment 3, we used the bat-and-ball problem and other CRT-like problems as materials to control intuition and analytical thinking more tightly by whether or not time pressure was given. This experiment was also conducted with Chinese university students to further explore the performance of the CRT with Chinese participants. 

### 4.1. Method

#### 4.1.1. Participants

Thirty-nine right-handed participants (17 males, mean age = 22.50 ± 1.94 years) from university were enrolled in the experiment. This experiment was approved by the local ethics committee of university. All participants provided written informed consent in accordance with the Declaration of Helsinki [47] and received a monetary reward for their participation in the experiment. After completing all data collection, participants were paid 15 CNY.

#### 4.1.2. Materials 

For the conflict conditions, we used the same materials provided in Experiments 2 and 3 in Boissin et al. (2021) [23], as follows:

*A bat and two balls cost $2.60 in total.*

*The bat costs $2 more than two balls.*

*How much does one ball cost?*

We also used modified versions of the bat-and-ball problem, which used quantities instead of prices.

Similarly, we used experimental materials with non-conflict conditions, as follows:

*In an office, there are 150 pens and pencils in total.*

*There are 100 pens.*

*How many pencils are there in the office?*

In the above example, the conflict condition requires the suppression of the intuitive answer ($0.30), and the correct answer ($0.15) is obtained after calculation. In contrast, the non-conflict condition only requires simple subtraction to get the correct answer (50 pencils). In addition, to prevent the participants from guessing our experimental purpose and producing a practice effect, we also set interference items, as follows:

*In an office, there are 150 pens and pencils in total.*

*There are 100 pens.*

*How many kinds of stationery are there in the office?*

We have listed 10 examples each of conflict, non-conflict, and interference conditions in the Supplementary Materials.

#### 4.1.3. Procedure

##### Pre-Experiment

Before the formal experiment, we recruited 19 college students (three males; mean age = 24.79 ± 2.75 years) to evaluate the time needed to read the questions. First, we presented the question stem (A bat and two balls cost $2.60 in total. The bat costs $2 more than the two balls.) and asked the participants to press the spacebar on the keyboard immediately after they finished reading and understanding the question. Because Raoelison et al. (2021) [25] set a time limit of 7000 ms to ensure that participants could read a question completely, we set the time limitation for this screen at 7000 ms. Then, the questions and answers (How much does one ball cost? $0.30/0.15) were presented, and participants were instructed to choose the correct answer. In this part of the experiment, we just calculated the reaction time for the two screens. The results indicated that participants needed, on average, 5187.56 ms (*SD* = 1004.16) to read and comprehend the question stem, and they needed 1648.08 ms (*SD* = 451.80) to read the problem and click on a response option. Hence, to ensure that most of the participants fully understood the question, the presentation time for the question stem was set to 6000 ms, and the time for the question was set to 1000 ms in the formal experiment. 

##### Formal Experiment

Another 39 college students (16 males; mean age = 23.63 ± 2.15 years) were recruited to participate in the formal experiment.

**Figure 1:**
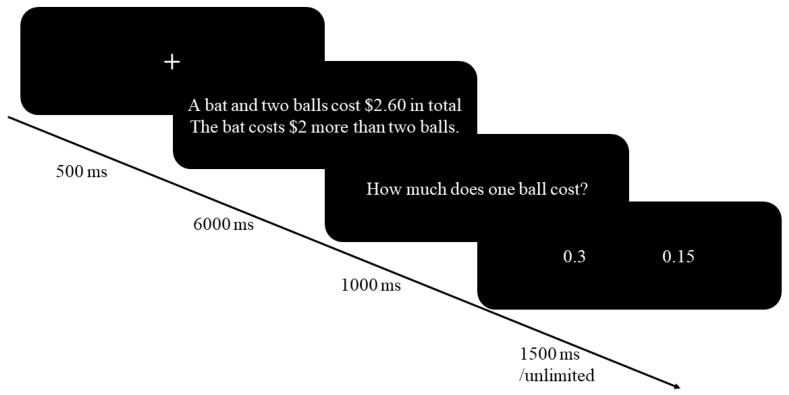
Illustration of an experimental trial in Experiment 3.

A mixed experimental design of 2 × 2 (conflict/non-conflict × limited/unlimited) was adopted, in which limited time and unlimited time were inter-group variables. The experiment was divided into two blocks, and each block had 60 trials. Each block contained 20 conflicting stimuli, 20 non-conflicting stimuli, and 20 interfering stimuli. Each trial was presented at random, and the positions of the answer were balanced. The difference between the limited group and the unlimited group was that the response time for the problem was set to 1500 ms and unlimited time, respectively. The experimental flowchart is shown in Figure 1.

Both the pre-experiment and formal experiment used E-prime 2.0 software (Psychology Software Tools, Pittsburgh, PA, USA) to complete the program and render it on the desktop computer. Participants were asked whether they had ever taken this test before at the end of the experiment.

### 4.2. Results

**Figure 2:**
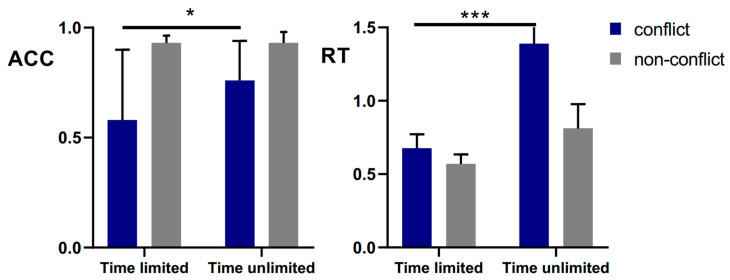
The accuracy and response time of time limited and unlimited groups (* *p* < 0.05; *** *p* < 0.001).

Figure 2 shows the descriptive statistics of the conflict and non-conflict groups. Because the non-conflict task was relatively simple, participants whose accuracy rate was lower than 0.7 in the non-conflict task were deleted and excluded from subsequent analysis. Finally, 36 participants (15 males, mean age = 22.5 ± 1.99 years) were included in the analysis. 

Due to the relatively small sample size in Experiment 3, we did not analyze gender as an independent variable, but included it as a covariate in subsequent analyses. We conducted a repeated measure analysis of variance (ANOVA) on accuracy with gender as a covariate, conflict (conflict vs. non-conflict) as a within-subjects factor, and time as (limited vs. unlimited) a between-subjects factor. The results showed that the main effect of conflict is not significant (*F* (1, 33) = 1.371, *p* = 0.250, η_p_^2^ = 0.040), the main effect of gender is not significant (*F* (1, 33) = 1.603, *p* = 0.214, η_p_^2^ = 0.046), and there is no main effect of time (*F* (1, 33) = 2.784, *p* = 0.105, η_p_^2^ = 0.078). The interaction between gender and conflict is not significant (*F* (1, 33) = 4.601, *p* = 0.229, η_p_^2^ = 0.044), and the interaction between conflict and time is significant, (*F* (1, 33) = 4.485, *p* = 0.039, η_p_^2^ = 0.122). The simple effect analysis shows that there is a significant difference between conflict and non-conflict under time-limited and unlimited conditions (*p*s < 0.001). Under conflict conditions, the difference between the time-limited and unlimited groups was marginal (*p* = 0.061), while under non-conflict conditions, the difference between the time groups was not significant (*p* = 0.355).

We then conducted a repeated measure ANOVA on response time with gender as a covariate, conflict (conflict vs. non-conflict) as the intra-group factor, and time (limited vs. unlimited) as the inter-group factor. The results revealed that the main effect of conflict is not significant (*F* (1, 33) = 0.055, *p* = 0.816, η_p_^2^ = 0.002), the main effect of gender is not significant (*F* (1, 33) = 0.082, *p* = 0.776, η_p_^2^ = 0.002), and the main effect of time is significant (*F* (1, 33) = 23.847, *p* < 0.001, η_p_^2^ = 0.419). The interaction between gender and conflict is not significant (*F* (1, 33) = 0.293, *p* = 0.592, η_p_^2^ = 0.009), and the interaction between conflict and time is significant (*F* (1, 33) = 10.524, *p* = 0.003, η_p_^2^ = 0.242). The simple effect analysis showed that there is a significant difference between time-limited and unlimited groups under conflict and non-conflict conditions (*p*s < 0.001). In the time unlimited group, there were significant differences between the conflict and non-conflict conditions (*p* < 0.001), while in the time-limited group, the difference between the conflict was not significant (*p* = 0.296).

### 4.3. Discussion

In Experiment 3, we investigated the CRT performance of Chinese college students by using the conflict and non-conflict problems of the CRT-liked problem and strictly controlling the intuition and analytical thinking of the participants by giving them time pressure or not. The results revealed that under the conflict condition, the accuracy rate under time-limited conditions was significantly lower than that under non-time-limited conditions, and no correlation was found with gender or grade. 

## 5. General Discussion

In this study, the CRT was analyzed through three experiments. We found that the average response accuracy for bat-and-ball problems was 68% when the participants were asked to answer an online questionnaire without taking too much time. In the offline experiments, participants were given pens and paper and asked to write down their answers, and the average accuracy was 87%. Finally, the reading time was strictly controlled, and time pressure was added to the answer time. The results showed that the response accuracy was 68% on average. Our study used Chinese college students to conduct systematic experimental verification of the CRT to confirm its universality. It is obvious that the response accuracy of Chinese college students is significantly higher than the results obtained in previous studies. We also found that individuals give significantly more intuitively incorrect answers under time pressure than under no time pressure.

First, the same experimental materials and instructions were used in experiment 1 and 2, but the accuracy rates were 68% and 87%, respectively. We believe that this was caused by the different experimental conditions, namely the experimental site. Hosseini et al. (2014) [48] compared the scores of Iranian first-year English majors on two tests and found that those who took the online test scored significantly lower than those who took the traditional test. Jeong (2014) [49] compared the Korean language and science test scores of sixth graders in Korea and found that all participants performed better on the traditional test. Our results were similar to these two studies in that the offline experiment results were better than the online test results. We suspect the cause of this phenomenon is that most participants are used to offline testing. That is, the participants in the laboratory were seated at a table and given a math questionnaire. Although the questionnaires were meant to measure intuition, this environment may have triggered memories of formal testing (like college entrance exams) among the Chinese participants. Furthermore, offline experiments require participants to physically come to the laboratory, which introduces situational pressure compared to unsupervised online experiments. Research has demonstrated that individuals may experience impaired cognitive and executive abilities when subjected to excessive stress [50]. Conversely, there is also research suggesting that moderate levels of stress can stimulate individuals to better concentrate their attention, thereby facilitating more effective handling of challenging tasks [51,52]. Participation in experiments is voluntary, so offline experiments can be considered as having a moderate level of pressure, thereby promoting analytical thinking and enhancing individual behavioral performance. These may have caused the participants to transition faster from intuitive thinking to analytical thinking. In other words, we could say that the situation increased participants’ “sensitivity to detecting intuitive traps”. Compared with offline CRT tasks, participants may prefer to answer interesting questions in online questionnaires.

Experiments 1 and 2, with relatively large sample sizes, both found a significant correlation between gender differences and CRT scores, which is consistent with previous studies. Studies with adults [4,53,54] found that males scored higher on the CRT than females, and females gave more intuitive responses than males. Campitelli and Gerrans (2014) [4] also showed that women struggled with inhibiting the intuitive response, especially in the case of the “bat and ball” problem. In Experiment 3, no significant correlation was observed between gender and CRT-liked, which might be due to the small sample size. In conclusion, from this perspective, it further validates that CRT is an effective measure for predicting intuitive and analytical thinking.

Under the time pressure of Experiment 3, the accuracy to conflict stimulus showed a significant difference. Time pressure is one way to strictly separate intuition from analytical thinking. Under time pressure, people adopt strategies to simplify information processing and response. That is, people search until they find a solution that meets their minimal needs and then adopt that solution without searching further [55]. High levels of time pressure lead to perceptual narrowing, which reduces the use of available cues, diminishes alertness, and decreases working memory capacity [56,57,58]. Under a time constraint, the operator may have neither the time nor the attentional resources to examine and evaluate multiple possible hypotheses. At the same time, the CRT causes people to fall into an intuitive thinking trap. Under time pressure, the proportion of intuitive wrong answers is significantly higher than that under no time pressure. Furthermore, the non-conflict condition is not affected by time-limited and time-unlimited conditions, which may be influenced by the characteristics of the materials in the conflict and non-conflict conditions. Firstly, the non-conflict materials do not have intuitive traps in their linguistic expression; secondly, the calculation method for the non-conflict condition is relatively simple. Therefore, even under time pressure, the responses in the non-conflict condition have reached a ceiling effect. The conflict condition, however, is different; the traps in the description and the relative complexity of the calculation pose a significant challenge to individuals. 

Our time-pressure study repeated previous results, which showed significant differences in the accuracy of intuitive and analytical thinking under conflict stimulus. Based on previous research, the accuracy rate of the CRT in Western countries is approximately 35% under non-time-limited conditions and about 15% under time-limited conditions [22,25,26]. The accuracy rate under time-limited conditions obtained in this study is 58%. Obviously, the accuracy rate of the CRT in Western countries is quite different from that of Chinese college students in this study. There has been substantial research on whether the CRT task assesses cognitive propensity or mathematical ability, and studies have shown that people find it difficult to solve these problems. Moreover, those who perform well on the CRT tend to perform well on numeracy tests and other general ability tests, and they tend to avoid biases in judgment and decision-making tasks [10,59,60]. In terms of math ability, many early studies have found that Asians have higher math ability than people in most Western countries [61]. Moreover, after controlling for education level and IQ, cross-cultural differences in arithmetic performance have remained significant [62,63]. This may be a result of the cultural disparities between China and Western nations, variations in parental upbringing, and the application of distinct educational strategies. Many researchers showed that the structure of the Chinese language gives children an advantage in fundamental mathematical abilities. For instance, they can identify pre-algebraic structures of writing from their activities in preschool [64,65]. Other studies also show that Asian parents, compared to parents from Western cultures, tend to strongly promote the development of good basic mathematics skills and a stronger epistemological discipline foundation [66,67]. The education system in China is content-oriented, exam-oriented, and highly competitive [68]. Empirical studies have shown that higher teaching quality by Chinese teachers and a greater emphasis on direct instruction in the classroom may help Chinese students outperform American students in math [69].

Although CRT does assess numerical ability to a certain extent [12,70], it mainly assesses individual differences in the tendency to use intuition/reflexes [1,2]. Supporters argue that solving the CRT “does not require high arithmetical skills” [71]; that is, if people think about CRT problems, even those with low mathematical ability can answer them correctly. Studies have shown that Chinese culture emphasizes the value of learning and the Chinese education system emphasizes achievement. Additionally, Chinese society adopts a content-intensive, test-oriented curriculum, so Chinese students learn by focusing on memorizing class material and practicing it repeatedly [72,73]. Chinese students growing up in this educational background may be sensitive to CRT problems, so they are better able to solve the problems from the formula.

In the context of Chinese education, students tend to memorize key points and focus on repeated practice and memorization in order to maximize test scores [66]. In the CRT without a time limit condition, the Chinese participants still achieved high response accuracy. Our study also shows that the CRT under time pressure is suitable for Chinese college students, which can also be an effective way to screen individuals with good logical intuition.

This study provides a preliminary exploration of the performance of Chinese college students in the CRT, but it has certain limitations. Firstly, the experimental design of Experiment 3 did not select two groups of subjects with different educational backgrounds but identical in other characteristics. Secondly, the lack of significant correlation between gender and CRT in Experiment 3 may be attributed to an insufficient sample size. However, the specific reasons necessitate further exploration. Future research could select a representative enough number of individuals from Western and Eastern cultures to conduct the same experimental design, further exploring the performance differences in the CRT under different educational backgrounds. This will contribute to a deeper understanding of how educational background influences the performance in the CRT.

## 6. Conclusions

Our study takes Chinese college students as participants to explore how they perform in cognitive reflection tests with a background of the Chinese education style. The study suggests that although Chinese participants have a higher accuracy rate, the CRT, which is prone to making people give incorrect intuitive responses under time pressure, is still applicable in China.

## Figures and Tables

**Table 1:** CRT studies.

Number	Research	Participant (Female)	Task	Accuracy	Time limited
1	Boissin et al. [23]	104	Two-response paradigm (bat and ball)	21.2% (pre-test) 17.2%/13.8% (study 1) 6.4%/15.3% (study 2)	Unlimited time to respond
2	Raoelison et al. [9]	123 (79)	Two-response paradigm	23.5% (slow)/19.9% (fast)	Intuition and 4 s/25 s
3	Janssen et al. [25]	50 (30)	Two-response paradigm	28.2%/27.7 (study 1) 15.1%/28.8% (study 2)	Intuition and 5 s
4	Raoelison et al. [26]	100	Two-response paradigm	9.6%/13% (study 1) 12%/13.7% (study 2)	Intuition and 5 s
5	Bago et al. [22]	231 (176) 143 (80) 140 (95)		27.3% 17.8% 23.8	Unlimited time to respond
6	Frey et al. [27]	248		21%	Unlimited time to respond
7	Johnson et al. [28]	313 (266)		21.6% (no load) 15.9% (low load) 3.3% (high load) 3.3% (extra high load)	Unlimited time but load
8	Pennycook et al. [5]	372 (268)	Cognitive reflection test	30.3%	/
9	Travers et al. [29]	131	Cognitive reflection test (3 item)	36%	/
10	Sirota and Juanchich [30]	452 (273)	Cognitive reflection test (7 item)	39.5%	/
11	De Neys et al. [21]	248	Cognitive reflection test (3 item)	21%	/

**Table 2:** The accuracy of each question in CRT task [M (*SD*)].

	Bat-and-Ball	Lily Pads	Machine
Experiment 1: ACC	0.685 (0.465)	0.418 (0.498)	0.610 (0.515)
Experiment 2: ACC	0.872 (0.349)	0.846 (0.622)	0.854 (0.353)

## Data Availability

The data that support the findings of this study are available upon request from the corresponding author. The data are not publicly available due to their confidential contents that could compromise the privacy of the research participants.

## Supplementary Materials

The following supporting information can be downloaded at https://www.mdpi.com/article/10.3390/bs14040348/s1.
