# Supplementary material for: Does the Cognitive Reflection Test Work with Chinese College Students? Evidence from a Time-Limited Study
Source: Behav Sci (Basel). 2024 Apr 22;14(4):348. doi: 10.3390/bs14040348 (PMC11047395; doi:10.3390/bs14040348)
Supplement: Supplementary file 1 [file behavsci-14-00348-s001.zip › behavsci-2927915-supplementary.pdf]

Conflict conditions (10):

1.A cheese and two breads cost a total of $2.8.
A cheese is $2 more expensive than two breads.
Question: How much does a bread cost?

2.An orange and two tangerines cost a total of $4.6.
An orange is $4 more expensive than two tangerines.
Question: How much does a tangerine cost?

3.A light bulb and two pillows cost a total of $3.4.
A light bulb is $3 more expensive than two pillows.
Question: How much does a pillow cost?

4.A necklace and two rings cost a total of $2.2.
A necklace is $2 more expensive than two rings.
Question: How much does a ring cost?

5.A table and two chairs cost a total of $5.4.
A table is $5 more expensive than two chairs.
Question: How much does a chair cost?

6.A keyboard and two mice cost a total of $2.8.
A keyboard is $2 more expensive than two mice.
Question: How much does a mouse cost?

7.A frying pan and two spatulas cost a total of $7.4.
A frying pan is $7 more expensive than two spatulas.
Question: How much does a spatula cost?

8.A grapefruit and two apples cost a total of $3.6.
A grapefruit is $3 more expensive than two apples.
Question: How much does an apple cost?

9.A toast and two egg tarts cost a total of $1.1.
A toast is $1 more expensive than two egg tarts.
Question: How much does an egg tart cost?

10.A pen and two pencils cost a total of $3.6.
A pen is $3 more expensive than two pencils.
Question: How much does a pencil cost?

Non-conflict conditions (10):

1.A store manager bought a total of 170 bananas and kiwis.
There are 100 bananas.
Question: How many kiwis are there?

2.A store is displaying 280 pianos and xylophones.
There are 100 pianos.
Question: How many xylophones are there?

3.There are 470 neurologists and botanists at the conference.
There are 400 neurologists.
Question: How many botanists are there at the conference?
4.A wood company bought 570 drills and saws.
There are 500 saws.
Question: How many saws does this company have?
5.A retail store is classifying a total of 180 oranges and lemons.
There are 100 oranges.
Question: How many lemons need to be classified?
6.There are 430 daisies and jasmine flowers by the lake.
There are 400 daisies.
Question: How many jasmine flowers are there by the lake?
7.In the city, people use a total of 650 small motorcycles and bicycles.
There are 600 small motorcycles.
Question: How many bicycles are there in the city?
8.In the grassland, scientists found 150 zebras and elephants.
There are 100 zebras.
Question: How many elephants are there in the grassland?
9.There are 260 kangaroos and peacocks in the zoo.
There are 200 kangaroos.
Question: How many peacocks are there in the zoo?
10.There are 320 peaches and pears in the fruit shop.
There are 300 peaches.
Question: How many pears are there in the fruit shop?

Interference questions (10):
1.A cheese and two breads cost a total of $2.8.
A cheese is $2 more expensive than two breads.
Question: How many characters are presented in the stem?
2.An orange and two tangerines cost a total of $4.6.
An orange is $4 more expensive than two tangerines.
Question: How many times does the word “orange” appear in the stem?
3.A light bulb and two pillows cost a total of $3.4.
A light bulb is $3 more expensive than two pillows.
Question: How many characters are presented in the stem?
4.A necklace and two rings cost a total of $2.2.
A necklace is $2 more expensive than two rings.
Question: How many times does the word “necklace” appear in the stem?
5.A table and two chairs cost a total of $5.4.

A table is $5 more expensive than two chairs.

Question: How many times does the letter ‘o’ appear in the stem?

6.A keyboard and two mice cost a total of $2.8.

A keyboard is $2 more expensive than two mice.

Question: How many types of electronic products are presented in the stem?

7.A frying pan and two spatulas cost a total of $7.4.

A frying pan is $7 more expensive than two spatulas.

Question: How many times does the letter ‘a’ appear in the stem?

8.A grapefruit and two apples cost a total of $3.6.

A grapefruit is $3 more expensive than two apples.

Question: How many types of fruits are presented in the stem?

9.A toast and two egg tarts cost a total of $1.1.

A toast is $1 more expensive than two egg tarts.

Question: What is the sum of the numbers that appear?

10.A pen and two pencils cost a total of $3.6.

A pen is $3 more expensive than two pencils.

Question: What is the sum of the numbers that appear?
